# Complete genome sequence of *Psychrobacter* sp. GTC18467 isolated from snake in Japan

**DOI:** 10.1128/mra.00340-25

**Published:** 2025-04-29

**Authors:** Masahiro Hayashi, Jun Yonetamari, Yoshinori Muto, Kaori Tanaka

**Affiliations:** 1 Institute for Glyco-core Research iGCORE, Gifu University 12785, Gifu, Gifu Prefecture, Japan; 2 Division of Anaerobe Research, Life Science Research Center, Gifu University 12785, Gifu, Gifu Prefecture, Japan; 3 Gifu University Center for Conservation of Microbial Genetic Resource 12785, Gifu, Japan; 4 Division of Clinical Laboratory, Gifu University Hospital 476117, Gifu, Gifu Prefecture, Japan; 5 United Graduate School of Drug Discovery and Medical Information Sciences, Gifu University 520344 , Gifu, Gifu Prefecture, Japan; Indiana University, Bloomington, Bloomington, Indiana, USA

**Keywords:** *Psychrobacter*, genomes

## Abstract

The genus *Psychrobacter* belongs to the family Moraxellaceae within the class *Gammaproteobacteria*. Here, we report the complete genome sequence of a new *Psychrobacter* species obtained from snake skin in Japan. The genome comprised a circular chromosome with a length of 2,642,444 bp and three circular plasmids.

## ANNOUNCEMENT

Identifying pet animal pathogens through genomic analysis is important for understanding pathogenic mechanisms and zoonotic infection risks (1, 2). We analyzed strain GTC18467, isolated from *Python regius* skin in Seki City, Gifu, Japan, in 2024. The strain was cultured on tryptic soy agar with sheep blood at 37°C for 48 h under aerobic conditions and was submitted to our laboratory as a presumptive *Psychrobacter* strain for further identification. Matrix-assisted laser desorption/ionization time-of-flight mass spectrometry (MALDI-TOF MS; Bruker, Bremen, Germany; 9607 MSPs) was used for identification, confirming its genus as *Psychrobacter*. The strain was stored at −80°C after four passages during identification.

The strain was cultured on tryptic soy agar under the same conditions. Colonies were scraped, suspended in TE buffer, and pelleted by centrifugation. Genomic DNA was extracted using NucleoBond HMW DNA (MACHEREY-NAGEL, Japan).

For identification, the 16s rRNA gene was sequenced after PCR amplification using primers 16S-8F (AGAGTTTGATCMTGGCTCAG) and 16S-1492R (GGYTACCTTGTTACGACTT). BLASTN analysis (3) identified the strain as *Psychrobacter* sp., most closely related to *Psychrobacter ciconiae* 176/10 (type strain Acc. no. NR_134796), with 97.4% similarity.

The genome was sequenced using PromethION 2i (Oxford Nanopore Technologies [ONT], Oxford, UK) for long-read sequencing and DNBSEQ (MGI Tech Co., Ltd., Shenzhen, China) for short-read sequencing (4–6). The same DNA extract was used for both sequencing processes. A library was constructed with a Native Barcode Sequencing Kit (SQK-NBD114-24; ONT) without shearing for long-read sequencing. Small DNA fragments were removed using Short Read Eliminator XS (Pacific Biosciences of California Inc., USA). Sequencing was performed on a PromethION 2i (ONT) with a FLO-PRO114M flow cell. Base calling was done with Dorado v.7.2.14 (ultra-high accuracy mode), and raw reads were trimmed and quality-filtered using NanoFilt v.2.7.1 (7) with parameters “-l 1000-q 10--headcrop 50.” For short reads, library construction was done with the MGIEasy FS DNA Library Prep Set (MGI Tech), and 2 × 150 bp paired-end sequencing was performed on the DNBSEQ-G400 platform (MGI Tech). Reads were processed with fastp v.0.20.1 (8) using “-q 30 -n 20 -t 1 -T 1.” Short-read quality was assessed using fastp (8), and long-read quality was scored using NanoPlot 1.32.1 (8). Short reads (with over 95.3% of bases >Q30) and long reads (mean read quality 15.8) were assembled using Unicycler v.0.4.8 (9) with default settings. Assembly circularization and rotation were performed automatically in Unicycler, and the assembly was rotated to start with the *dnaA* gene on the forward strand. The contig graph was visualized with Bandage v.0.8.1 (10), and the taxonomic integrity was confirmed using blobtools v.1.0 with short reads (11).

Table 1 summarizes the genomic information. Unicycler and Bandage indicated a complete circular genome. Quality and genome statistics were computed using CheckM (v1.2.2) (12) and seqkit (v2.9.0) (13). CheckM confirmed the genome was 100% complete with 0% contamination. The DDBJ Fast Annotation and Submission Tool (14) predicted 2,275 coding sequences, 12 rRNAs, 48 tRNAs, and 9 CRISPR sequences. Species identification using an average nucleotide identity value of the genome sequence with GTDB-tK (v.2.3.2) was unsuccessful (15).

**TABLE 1:** Information regarding the complete genome sequence of a *Psychrobacter* sp. strain isolated from a snake in Japan

	Strain name
Parameter	*Psychrobacter* sp. GTC18467
DNBSEQ sequencing^*a*^	
No. of reads	4,772,296
Size (kb)	715,844
Avg coverage (x)	271
DRA accession no.	DRR623152
ONT sequencing^*a*^	
No. of reads	136,869
Size (kb)	1,494,621
Avg read length (bp)	10,920
Avg coverage (x)	566
N_50_	14,570
DRA accession no.	DRR623151
Assembly	
Assembly N50 (bp)^*c*^	2,598,574
Estimated genome completeness (%)^*c*^	100.0
Estimated genome contamination (%)^*c*^	0.0
Genome structure	One chromosome and three plasmids
DDBJ/GenBank accession no.	AP038920 (GTC18467) AP038921 (pGTC18467-1) AP038922 (pGTC18467-2) AP038923 (pGTC18467-3)
Genome size (bp)	2,598,574 (GTC18467) 26,064 (pGTC18467-1) 12,994 (pGTC18467-2) 4,812 (pGTC18467-3)
GC content (%)	
(Chromosome/plasmid name)	45.34 (GTC18467) 41.29 (pGTC18467-1) 39.85 (pGTC18467-2) 42.21 (pGTC18467-3)
Average read depth	
	259 (GTC18467) 1,037 (pGTC18467-1) 778 (pGTC18467-2) 1,528 (pGTC18467-3)
No. of coding sequences^*b*^	2,275
Number of rRNAs^*b*^	12
Number of tRNAs^*b*^	48
Number of CRISPRs^*b*^	9

^*a*^ DRA, DDBJ Sequence Read Archive.

^*b*^ DFAST, DDBJ Fast Annotation and Submission Tool.

^*c*^ Determined with seqkit v2.9.0.

## Data Availability

The genome sequences of the *Psychrobacter* sp. GTC18467 strain from the snake have been deposited in DDBJ (https://www.ddbj.nig.ac.jp/index.html) under the accession numbers AP038920–AP038923. Raw sequence data for GTC18467 have been deposited in the DDBJ/Sequence Read Archive under accession numbers DRR623151 and DRR623152.
